# Nuclear Receptor 4A2 (NR4A2/NURR1) Regulates Autophagy and Chemoresistance in Pancreatic Ductal Adenocarcinoma

**DOI:** 10.1158/2767-9764.CRC-21-0073

**Published:** 2021-11-03

**Authors:** Mehrdad Zarei, Rupesh Shrestha, Sneha Johnson, Zuhua Yu, Keshav Karki, Ali Vaziri-Gohar, Jessica Epps, Heng Du, Larry Suva, Mahsa Zarei, Stephen Safe

**Affiliations:** 1 Department of Surgery, University Hospitals; Case Western University, School of Medicine, Cleveland, OH.; 2 Department of Biochemistry and Biophysics, Texas A&M University, College Station, TX.; 3 Department of Veterinary Physiology and Pharmacology, Texas A&M University, College Station, TX.; 4 Henan University of Science and Technology, Luoyang, Henan Province, China, P.R.; 5 Department of Integrative Biosciences, College of Veterinary Medicine, Texas A&M University, College Station, TX.; 6 Department of Medicine, Brigham and Women's Hospital and Harvard Medical School, Boston, MA.

## Abstract

Pancreatic ductal adenocarcinoma (PDAC) is a highly aggressive cancer with poor prognosis and chemotherapy with gemcitabine has limited effects and is associated with development of drug resistance. Treatment of Panc1 and MiaPaca2 pancreatic cancer cells with gemcitabine induced expression of the orphan nuclear receptor 4A2 (NURR1) and analysis of The Cancer Genome Atlas indicated the NURR1 is overexpressed in pancreatic tumors and is a negative prognostic factor for patient survival. Results of NURR1 knockdown or treatment with the NURR1 antagonist 1,1-bis(3΄-indolyl)-1-(p-chlorophenyl)methane (C-DIM 12) demonstrated that NURR1 was prooncogenic in pancreatic cancer cells and regulated cancer cell and tumor growth and survival. NURR1 is induced by gemcitabine and serves as a key drug resistance factor and is also required for gemcitabine-induced cytoprotective autophagy. NURR1-regulated genes were determined by RNA sequencing of mRNAs expressed in MiaPaCa2 cells expressing NURR1 and in CRISPR/Cas9 gene–edited cells for NURR1 knockdown and Kyoto Encyclopedia of Genes and Genomes enrichment analysis of the differentially expressed genes showed that autophagy was the major pathway regulated by NURR1. Moreover, NURR1 regulated expression of two major autophagic genes, *ATG7* and *ATG12*, which are also overexpressed in pancreatic tumors and like NURR1 are negative prognostic factors for patient survival. Thus, gemcitabine-induced cytoprotective autophagy is due to the NURR1–ATG7/ATG12 axis and this can be targeted and disrupted by NURR1 antagonist C-DIM12 demonstrating the potential clinical applications for combination therapies with gemcitabine and NURR1 antagonists.

**Significance:**

Gemcitabine induces NURR1-dependent ATG7 and ATG12 cytoprotective autophagy in PDA cells that can be reversed by NURR1 antagonists.

## Introduction

Pancreatic ductal adenocarcinoma (PDAC) is a lethal human malignancy with a five-year survival rate of 9% and it is estimated that by 2030 pancreatic cancer will be the second leading cause of cancer-related deaths in United States (1). Although new therapeutic regimens against PDAC have improved treatment of this disease, the late-stage detection, lack of targeted therapies, and chemoresistance are still some of the major challenges for enhancing patient outcomes. The current treatments include surgical resection, which is followed by adjuvant chemotherapy (2) and palliative chemotherapy [with gemcitabine or FOLFIRINOX (folinic acid, 5-flouorouracil, irinotecan and oxaliplatin)] (3–5). Gemcitabine (Gemzar; 2′,2′-difluorodeoxycytidine), an FDA approved chemotherapeutic drug, is used for treatment of different cancers and overall survival and progression-free survival is enhanced by gemcitabine compared with those receiving non–gemcitabine-based therapy (6). Although, these treatments are initially effective in increasing survival of patients with pancreatic cancer, residual neoplastic cells usually result in relapse and subsequent appearance of more aggressive and lethal tumors (7). The success of cancer treatments for patients with PDA largely depends on diagnosis during initial stages of the disease. Cellular mechanisms such as apoptosis, necroptosis, and autophagy also determine the advancement and response to these cancer treatments (8). Thus, to find a remedy for treating this devastating disease, it is important to understand the mechanism through which pancreatic cancer cells acquire resistance to current therapeutics.

*NURR1* (NR4A2) is an orphan nuclear receptor and a transcription factor that activates target genes by binding as monomers or dimers to cognate *cis*-elements in target gene promoters (9). *NURR1* has been characterized as an important regulator in neuronal development and there is also increasing evidence of a prooncogenic role for their receptor in solid tumors (9–11) *NURR1* regulates cell growth survival and metabolism (12–14) and studies in this laboratory identified 1,1-bis (3′-indolyl)-1-(p-chlorophenyl) methane (C-DIM12) as prototypical *NURR1* ligand (15, 16). A recent study showed that C-DIM12 inhibited glioblastoma cell and tumor growth and also blocked *NURR1*-dependent prooncogenic pathways in glioblastoma (15). It was previously reported that induction of *NURR1* promotes 5-fluorouracil (5-FU) resistance in squamous cell carcinoma (17); however, the mechanism of *NURR1*-mediated chemoresistance and its role in pancreatic cancer has not been determined.

Autophagy is an evolutionary cellular response to diverse stress and physiologic conditions during cancer progression and has been reported to induce tumor cell survival and drug resistance in cancer cells (18, 19). Autophagy is initiated with the formation of double layer membrane around the cytoplasmic components known as autophagosome which fuses with lysosomes to recycle these components for protein synthesis and energy production. It is closely regulated by a highly conserved set of genes known as autophagy-related genes (*ATG*; ref. 18). A recent study demonstrates that autophagy regulates the unique properties of cancer stem cells, such as differentiation and self-renewal, which contributes to tumor metastasis, tumor reoccurrence, and chemoresistance (20). A study, combining an autophagy inhibitor with photodynamic therapy, significantly reduced the colorectal tumor size suggesting that autophagy is a cytoprotective process (21), and in another study autophagy inhibition increased the drug sensitivity (22). Thus, understanding the mechanism of autophagy and drug resistance is an unsolved problem and could lead to enhanced therapeutic efficacy.

This study demonstrates that *NURR1* is essential for chemotherapeutic agent–induced cytoprotective autophagy through transcriptional regulation of ATGs. We show for first time that *ATG7* and *ATG12* are *NURR1*-regulated genes, and high expression of *NURR1*/*ATG7*/*ATG12* corresponds to the poor survival and prognosis of patients with PDA and the *NURR1*-*ATG7*/*ATG12* axis can be targeted by *NURR1* antagonists.

## Materials and Methods

### Cell Lines and Transfection with siRNA and Plasmids

MiaPaCa2 and Panc1 pancreatic cancer cell lines were obtained from ATCC and were cultured in in DMEM supplemented with 10% FBS (Gibco/Invitrogen), 1% l-glutamine (Gibco/Invitrogen), and 1% penicillin–streptomycin (Invitrogen) at 37°C in 5% humidified CO_2_ incubators. CRISPR/Cas9–mediated knockout of NURR1 in MiaPaCa2 cells was accomplished using guide RNAs targeting *NURR1*, fused with CRISPR/Cas9 and GFP protein (23). CRISPR Universal Negative Control plasmid (CRISPR06–1EA) was purchased from Sigma-Aldrich. Cells were harvested after 48 hours of transfection and GFP-positive cells were single sorted by using FACSCalibur flow cytometer. The guide RNA sequences used were:

*NURR1*–1(gatcccgggtcgtcccacat),
*NURR1*–2(gggcttgtagtaaaccgacc),

For all cell culture experiments, *Mycoplasma* testing (MycoAlert Mycoplasma Detection Kit, Lonza) was performed after each thawing and at least monthly. Cells were also authenticated using short tandem repeat analysis (Biosynthesis)

Cells were plated at 60% confluency in 6-well plates, and transient siRNA transfections (1 μmol/L) were performed using Lipofectamine 3000 (Invitrogen) and Opti-MEM (Invitrogen) according to the manufacturer's protocol; 48 hours after transfection, cells were treated or analyzed, as described previously (24, 25). siRNA oligos were purchased from Life Technologies (si*NURR1*, 4427038; si*ATG7*, 4392420; si*ATG12*, 4392420; siCTRL, AM4635).

### Cell Growth Assays

Cells were plated in 96-well plates at 1 × 10^3^ cells per well. After 5 days of incubation, cell growth was measured using Quant-iT PicoGreen dsDNA Assay Kit (Invitrogen). To estimate cell death, cells were trypsinized and counted after Trypan blue staining (Invitrogen) with a Hausser bright-line hemocytometer (Thermo Fisher Scientific; ref. 26). Annexin V/PI staining was performed using the Dead Cell Apoptosis Kit (Thermo Fisher Scientific, #V13245), according to the manufacturer's instructions. Staining was measured with an Accuri C6 flow cytometer and analyzed with FlowJo Version 10.2 software (26).

### Immunoblot Analysis

Cells were lysed using 1% Triton in TBS containing protease and phosphatase inhibitors. Tumors were lysed using 1× RIPA buffer containing protease and phosphatase inhibitors. Equal amounts of total protein were separated by electrophoresis on a 4%–12% Bis-Tris gel and transferred to a polyvinylidene difluoride membrane (23). Blots were blocked in 5% BSA, and then probed with antibodies against anti-*NURR1* (sc-376984, Santa Cruz Biotechnology), anti-*ATG* 7 (10088–2AP, ProteinTech), anti-*ATG* 12(D88H11, Cell Signaling Technology), anti-LC3BI/II (3868, Cell Signaling Technology), anti-cleaved PARP (9532S, Cell Signaling Technology), and anti-α-tubulin (Sigma). Chemiluminescent (32106, Thermo Fisher Scientific) signal was captured using a Syngene G-BOX iChemi XT imager (26).

### Clonogenic Assay

1000–2000 cells per well were plated in a 6-well plate. The media were not changed during experiments unless indicated. Upon completion of the experiments, colonies were fixed in reagent containing 80% methanol and stained with 0.5% crystal violet. To determine relative growth, dye was extracted from stained colonies with 10% acetic acid and the associated absorbance measured at 600 nm using a Microplate Reader/Synergy HT BioTek plate reader (23, 27).

### Luciferase Reporter Assays

Cells were seeded at 60% confluency in 6-well dishes, then transfected using lipofectamine 3000 (Thermo Fisher Scientific) with 2 μg of the promoter dual reporter plasmid (pCheck2–promoter *ATG7* [TSS = 11272324; Upstream = 1265, Downstream = 295; Length = 1561] or *ATG12* [TSS = 115841851; Upstream = 1271, Downstream = 228; Length = 1500]), purchased from GeneCopoeia (23). The luciferase activity of the cultured supernatant was measured 48 hours after transfection using Luciferase Assay Reporter Kit (Promega) according to the manufacturer's instructions (23).

### Electron Microscopy

Cells were cultured in permanox petri dish and treated with gemcitabine (1 μmol/L) and C-DIM12 (15 μmol/L) for 24 hours. The cells were fixed with 2.5% paraformaldehyde, 2% glutaraldehyde, 0.1 mol/L cacodylate buffer, and embedded using Epon 812. The ultra-thin sections (∼100 nm) were cut using a Leica EM UC6 ultramicrotome and diamond knife. The sections were then placed on copper grids, poststained with saturated Uranyl Acetate and Reynolds Lead Citrate, and imaged using an FEI Morgagni 268 transmission electron microscope equipped with a MegaView III CCD camera.

### Quantitative RT-PCR

Total RNA was extracted using RNAeasy Kit (Qiagen). cDNA was synthesized from 1 μg of total RNA using TagMan probes (NURR1, Hs00428691, Hs01117525; ATG7, Hs00893766, Hs04969948; ATG12, Hs00740818, Hs01047860; 18s, 99999901; Actin, Hs00157387, Life Technologies) and MultiScribe Reverse Transcriptase (Life Technologies). qRT-PCR analysis was performed using the Applied Biosystems 7500 Fast Real-Time PCR System (Life Technologies) and TagMan RT-PCR Master Mix (Life Technologies).

### RNA Sequencing

RNA quality was assessed via the Agilent 2100 Bioanalyzer (Agilent Technologies). Strand-specific RNA-sequencing (RNA-seq) library was prepared using NEBNext Ultra II Directional RNA Library Prep Kit (NEB) according to the manufacturer's protocols (27). RNA-seq was performed using 150-bp paired-end format on a NovaSeq 6000 (Illumina) sequencer. RNA-seq quality was checked by running FastQC, and TrimGalore was used for adapter and quality trimming (27). Sequence reads were aligned to the hg19 human genome build using the STAR aligning program (28). Quantification of all genes and their isoforms was performed using FPKM normalized values using Cufflinks v2.2.1, DESeq2 analysis with an *P*_adj_ < 0.05 was used to get a list of differentially expressed genes (29).

### Immunofluorescence Assay

Cells were seeded in 24-well plate (50,000 cells/well). After 24 hours, the media were discarded and the cells were then washed with 1× PBS. The cells were then fixed with 10% formaldehyde and permeabilized with 0.1% Triton-X 100. The cells were then incubated with primary antibody, overnight at 4°C and then with secondary antibody (Thermo Fisher Scientific #A11001 and Cell Signaling Technology #4412S)] for 2 hours at room temperature. The images were captured using Zeiss Imager.Z1 AXIO at 40× magnification.

### Chromatin Immunoprecipitation Assay

Cells were cross-linked for 10 minutes at room temperature by the addition of one-tenth volume of 11% formaldehyde (11% formaldehyde, 50 mmol/L HEPES pH 7.4, 100 mmol/L NaCl, 1 mmol/L EDTA pH 8.0, 0.5 mmol/L EGTA pH 8.0), followed by 5-minute quenching with 1/20th volume of 2.5 mol/L glycine. Cells were washed twice with PBS, with spins after each rinse, the supernatant was aspirated, and the cell pellet was flash frozen in liquid nitrogen. Frozen crosslinked cells were stored at −80°C (30).

Crosslinked cells were lysed with lysis buffer 1 (50 mmol/L HEPES pH 7.5, 140 mmol/L NaCl, 1 mmol/L EDTA, 10% glycerol, 0.5% NP-40, and 0.25% Triton X-100), pelleted, and resuspended in lysis buffer 2 (10 mmol/L TrisHCl pH 8.0, 200 mmol/L NaCl, 1 mmol/L EDTA, 0.5 mmol/L EGTA), and pelleted again (30). The pellet was resuspended in sonication buffer (50 mmol/L HEPES pH 7.5, 140 mmol/L NaCl, 1 mmol/L EDTA pH 8.0, 1 mmol/L EGTA, 0.1% sodium deoxycholate, 0.1% SDS, and 1% Triton X-100), and sonicated (30) using a Branson Sonifier (power setting 5) for 10 cycles at 30 seconds each on ice (18–21 W) with 60 seconds on ice between cycles.

Dynal magnetic beads (Sigma) (50 μL) were blocked with 0.5% BSA (w/v) in PBS, and then bound with 10 μg of antibody against *NURR1*, Abcam (AB41917). Sonicated crosslinked lysates were incubated overnight at 4°C with magnetic beads bound with antibody. Beads were pelleted, and then washed several times: two times with sonication buffer, one time with sonication buffer with 500 mmol/L NaCl, one time with LiCl wash buffer (10 mmol/L TrisHCl pH 8.0, 1 mmol/L EDTA, 250 mmol/L LiCl, 0.5% NP-40, 0.5% sodium deoxycholate) and one time with TE (10 mmol/L TrisHCl pH 8.0, 1 mmol/L EDTA). Bound protein and cross-linked DNA were eluted in elution buffer (50 mmol/L TrisHCl pH 8.0, 10 mmol/L EDTA, 1% SDS), and cross-links were reversed by overnight incubation using RNase A (10 mg/mL) and Proteinase K (20 mg/mL) for 1 hour, respectively, and DNA was purified with phenol/chloroform extraction and ethanol precipitation and used for qPCR (primers summarized in Supplementary Table S1).

### Mouse Studies

All experiments involving mice were approved by the Texas A&M University's Animal Care and Use Committee. Six-week-old, female, athymic nude mice (Nude-Foxn1nu) were purchased from ENVIGO. MiaPaCa2 (ctrl), CTRL.KO, *NURR1*.KO cells, were prepared in 100 μL solution comprised of 70% DPBS and 30% Matrigel. Suspensions of 3 × 10^6^ cells were then injected subcutaneously into the left and right flanks of mice. Tumor volumes were measured three times per week using calipers (Volume = Length × Width^2^/2), along with body weight. Mice with established tumors (after 25 days, mean tumor volume of ∼100 mm^3^) were randomly divided into four groups, which were then treated with vehicle (20 μL/g of 0.9% NaCl), gemcitabine (5 mg/mL, prepared in vehicle solution) 100 mg/kg intraperitoneally i.p.) twice biweekly, C-DIM12 (30 mg/kg i.p.; Monday/Wednesday/Friday; LC Laboratories #R-5000) or a combination of both. Upon termination of mouse experiments, mice were euthanized using carbon dioxide inhalation followed by cervical dislocation, and tumors were harvested.

### Statistical Analysis

Data were expressed as mean ± SEM of at least three independent experiments. An unpaired, two-tailed Student *t* test was used to determine the differences between groups (* *P* < 0.05; ** *P* < 0.01; *** *P* < 0.001). ANOVA test was used for the analysis of tumor measurements among treated groups.

### Data Availability

RNA-seq files have been deposited in Gene Expression Omnibus (GEO) with accession number GSE159099.

## Results

### Prognostic Significance of NURR1 in Patients with PDAC

**FIGURE 1:**
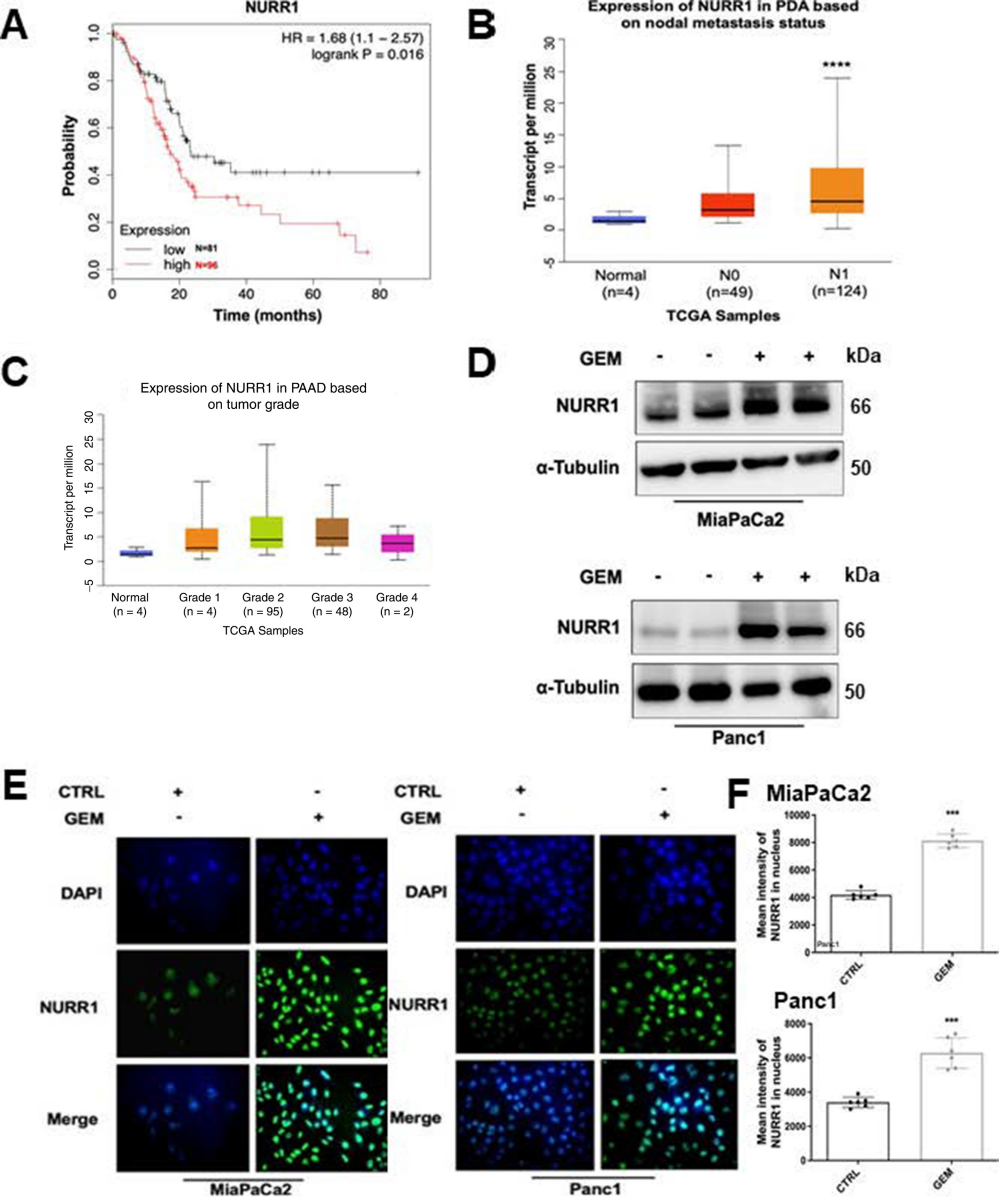
*NURR1* confers to PDA chemoresistance. **A,** TCGA research network for PDA database was used for expression of *NURR1* mRNA and correlated with patient's disease-free survival by using Kaplan–Meier survival analysis with log-rank tests (*P* < 0.016). **B,***NURR1* mRNA expression levels in patients with PDA based on nodal metastasis status. For each boxplot, median expression value, and 1st and 3rd quartiles are indicated (****, *P* < 0.00001). **C,***NURR1* mRNA expression levels in patients with PDA based on tumor grade. **D,** Immunoblot analysis of *NURR1* protein lysates from MiaPaCa2 and Panc1 cells after 48 hours of treatment with gemcitabine (GEM; 1 μmol/L). β-Tubulin serves as a loading control (three independent experiments performed). **E,** Immunofluorescence staining of MiaPaCa2 and Panc1cells after 24 hours of treatment with gemcitabine (1 μmol/L) *NURR1*, Alexa Fluor 488 (green); nucleus, DAPI. Merged image, nuclear localization of *NURR1* (three independent experiments performed, and at least 10 images per slide were analyzed for each condition). Magnification, 40×. **F,** Bars represent the quantification of mean intensity of *NURR1* in the nucleus. Each data point represents the mean ± SEM of three independent experiments (***, *P* < 0.001).

Previous studies showed that NURR1 is highly expressed in glioblastoma (15) and analysis of the published The Cancer Genome Atlas (TCGA) database showed that *NURR1* was also more highly expressed in tumor samples from a patient with PDA. Kaplan–Meier analysis of NURR1 expression data showed that NURR1 overexpression of PDA patients’ tumor samples was also significantly associated with their poor survival (Fig. 1A). Lymph node metastasis was studied in 173 patients with PDA and *NURR1* expression was significantly higher in patients with N0 and N1 PDA compared with normal individuals (Fig. 1B). *NURR1* was also overexpressed in grade 2 tumor samples confirming that high levels of *NURR1* are associated with the severity of PDAC (Fig. 1C).

PDA is a prime example of a tumor that develops chemoresistance and to investigate the possible relationship between chemotherapeutic drugs and *NURR1* expression pancreatic cancer cells were treated with gemcitabine and analysis by immunoblot and immunofluorescence showed that gemcitabine induced expression of *NURR1* in both MiaPaCa2 and Panc1 cells (Fig. 1D and E). This demonstrates that *NURR1* expression is increased during chemotherapeutic treatment and the mechanisms of gemcitabine induction of NURR1 and GEM – C-DIM12 interactions are unknown and are currently being investigated.

### NURR1 has a Cytoprotective Role Against Chemotherapeutic Drug–Induced Cell Death in Pancreatic Cancer Cells

**FIGURE 2:**
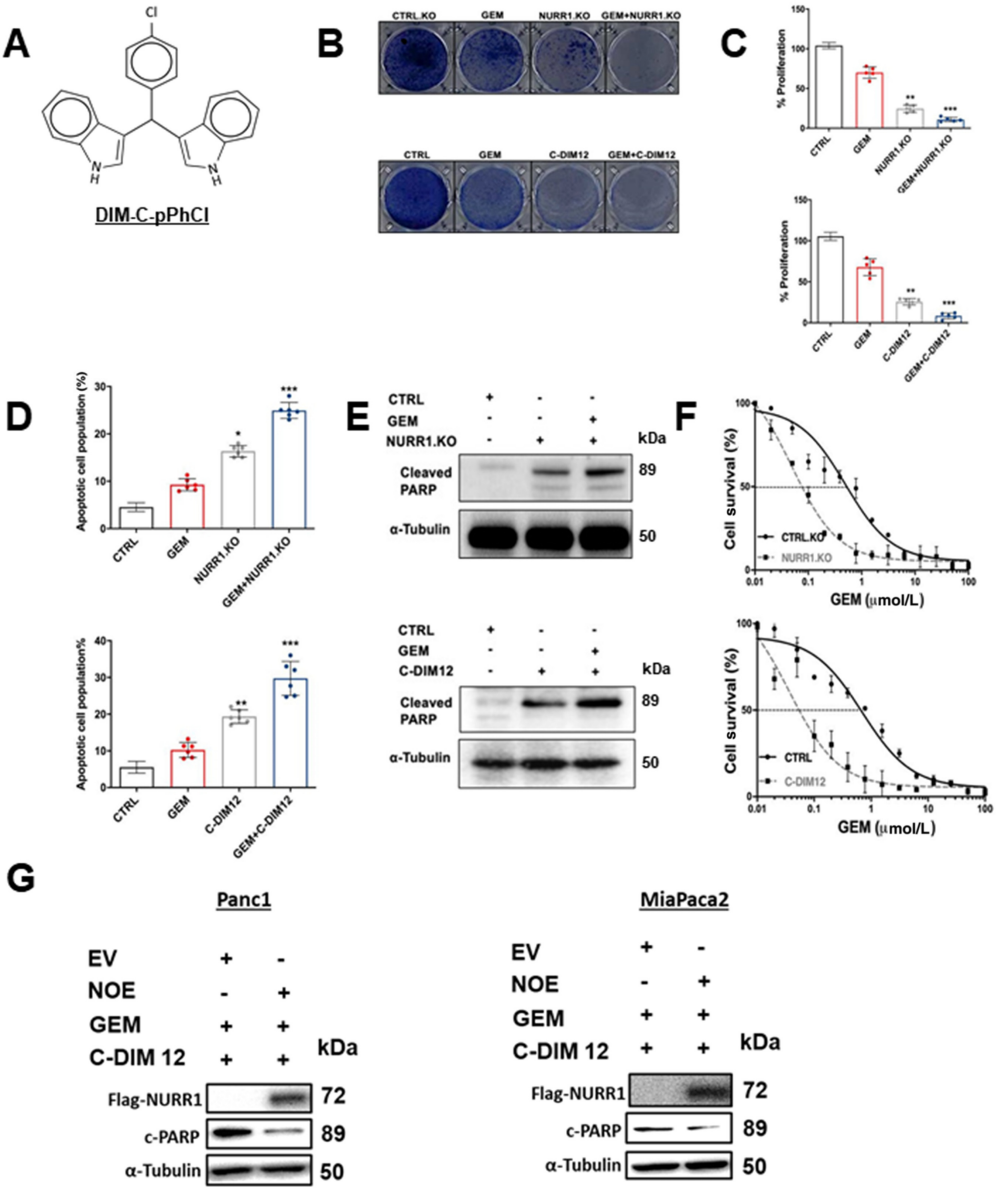
*NURR1* plays a cytoprotective role in pancreatic cancer. **A,** Chemical structure of DIM-C-pPhCl (C-DIM12). **B,** Images of crystal violet stained dishes that were treated with vehicle control, gemcitabine (GEM), C-DIM12, or NURR1 loss for 10 days after plating 1,000 cells per well in a 12-well plate (three independent experiments performed). **C,** Quantification of crystal violet staining is shown. Error bars indicate SEM of triplicate wells from a representative experiment (three independent experiments performed; **, *P* < 0.01; ***, *P* < 0.001). **D,** Apoptotic cell fraction was determined after treatment control (vehicle or CTRL.KO), gemcitabine, C-DIM12 or *NURR1* loss, or a combination of both (gemcitabine + C-DIM12/ gemcitabine +*NuRR1*.KO for 72 hours. Apoptotic cell death was quantified by Annexin V/propidium iodide (PI) staining and flow cytometry and is shown as the percentage of cells that were PI positive. Each data point represents the mean ± SEM of three independent experiments. *, *P* < 0.05; **, *P* < 0.01; ***, *P* < 0.001). **E,** Immunoblot analysis of cleaved PARP in control, gemcitabine, C-DIM12, or NURR1 loss treated cells with α-tubulin as a loading control in MiaPaCa2. **F,** Drug sensitivity was measured by PicoGreen DNA quantitation, in MiaPaCa2 cells, under the indicated culture conditions, and with varying doses of gemcitabine. Each data point represents the mean of four independent measurements. **G,** Panc1 MiaPaca2 cells were treated as indicated in the presence or absence of NOE or expression of empty vector (EV) and whole-cell lysates were analyzed by Western blots as outlined in the Materials and Methods.

To study the role of *NURR1* in drug resistance, we treated the MiaPaCa2 cells with the *NURR1* antagonist C-DIM12 (DIM-C-pPhCl; Fig. 2A) and gemcitabine and in parallel studies we also determined the effects of gemcitabine alone and after NURR1 knockout (by RNA interference) in MiaPaCa cells. C-DIM12 has been characterized as an NURR1 antagonist in cancer cells and has been used as a model compound for studying the actions of NURR1 (15, 16). Crystal violet assay, phase contrast microscopy, and Annexin V staining showed that gemcitabine and C-DIM12 alone were effective; however, treatment with gemcitabine in combination with C-DIM12 was a more potent inhibitor of cell proliferation and survival than the individual compounds alone (Fig. 2B–D) and similar results were observed in Panc1 cells (Supplementary Figs. S1A – S1D). Moreover, treating the cells with C-DIM12 in combination with gemcitabine increased apoptosis as determined by cleaved PARP in MiaPaCa2 (Fig. 2E) and Panc1 cells (Supplementary Fig. S2A and S2B). Results summarized in Fig. 2E and 2F also show that cleaved PARP (marker of apoptosis) and inhibition of cell growth by gemcitabine were enhanced by cotreatment with C-DIM12 or by *NURR1* knockout using CRISPR/Cas9 gene–edited cells and this response can be attenuated by overexpression of Nurr1 (NOE; Fig. 2G). Electron microscopy images also demonstrate that pancreatic cancer cells were more sensitive to gemcitabine when treated in combination with C-DIM12 and this is evidenced by enhanced cell shrinkage and nuclear condensation in treated compared with control cells. We also observed progressive fragmentation and increase in number of apoptotic bodies, which are indicators of apoptosis in the cells treated with gemcitabine and C-DIM12 (Supplementary Fig. S1C).

### ATG7 and ATG12 are the Key Targets of NURR1

**FIGURE 3:**
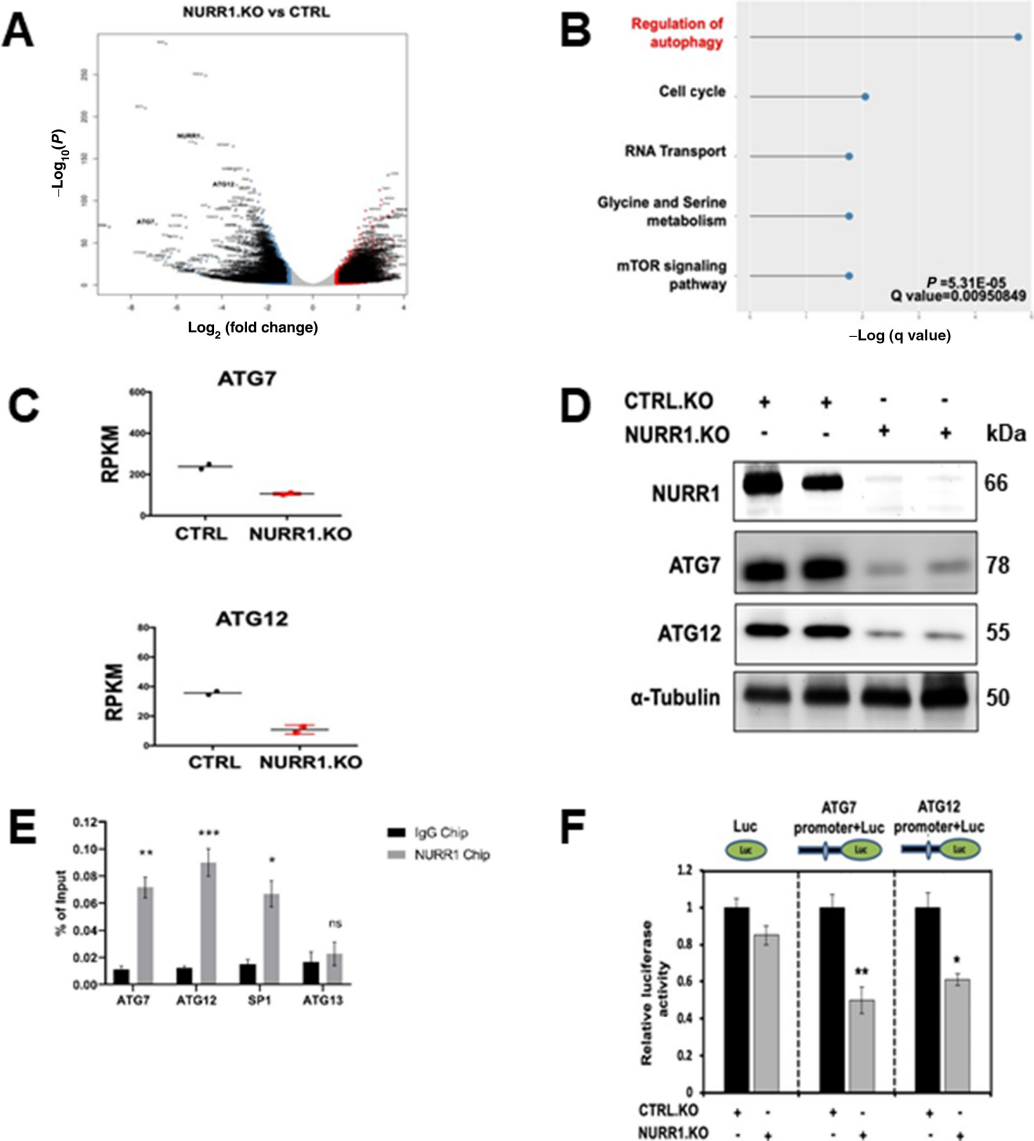
*ATG7* and *ATG12* are the key targets of *NURR1.***A,** Volcano plots of log_2_ fold change versus −log_10_(*P*) of RNA-sequencing data for *NURR1*.KO versus control (CTRL), for MiaPaCa2 cells subject to these manipulations. **B,** KEGG enrichment pathway analysis of NURR1-regulated pathways by comparing gene expressed wild-type MiaPaca2 cells and NURR1 knockout cells. **C,** qRT-PCR analysis of *ATG7*and *ATG12* expression in CTRL and *NURR1.KO* cells. Gene expression is normalized to β-actin. Each data point represents the mean ± SEM of four independent experiments (**, *P* < 0.01; ***, *P* < 0.001). **D,** Immunoblot analysis of *NURR1*, *ATG7*, and *ATG12* in *NURR1*.KO cells compared with CTRL MiaPaCa2 cells with α-tubulin as loading control. **E,** qPCR analysis of a ChIP assay in MiaPaCa2 cells shows a significant increase of NURR1 interactions on the in *ATG7* and *ATG12* promoters relative to IgG (**, *P* < 0.01; ***, *P* < 0.001). Results are expressed as the fold enrichment over input DNA. Error bars represent the mean ± SEM of three independent experiments. **F,** Dual luciferase assay of MiaPaCa2 cells with empty vector control (EV) and *NURR1.KO*. Cell lines were transfected with *Renilla* luciferase reporter constructs fused with *ATG7* or *ATG12* promoter, as well as a constitutive firefly luciferase expression construct for 24 hours. *Renilla* luciferase activity was normalized to firefly luciferase activity, and results shown are the average of four experiments ± SEM (*, *P* < 0.05; **, *P* < 0.01).

*NURR1* regulates specific gene activity and mediates cell survival, migration, invasion, and transformation (9–13, 15, 31). To identify *NURR1*-regulated genes that may be involved in drug resistance, we performed RNA-seq and Kyoto Encyclopedia of Genes and Genomes (KEGG) enrichment pathway analysis in MiaPaCa2 cells, modified by CRISPR/Cas9 gene editing for *NURR1* expression and in control cells expressing *NURR1* (Fig. 3A). To investigate pathways altered by the *NURR1* knockout, we performed KEGG enrichment pathway analysis and observed that regulation of genes associated with autophagy was enriched (Fig. 3B; Supplementary Table S2). The expression of the autophagic *ATG7* and *ATG12* genes (18) was downregulated in *NURR1*.KO cells when compared with *NURR1*.CTRL cells (Fig. 3A–C) and therefore the potential roles of *ATG7* and *ATG12* in *NURR1*-mediated chemoresistance was further investigated because autophagy plays a key role in cell survival and drug resistance (32). Immunoblot analysis confirmed that *NURR1* regulates the expression of *ATG7* and *ATG12* because knockdown of *NURR1* significantly downregulated levels of *ATG7* and 12 proteins (Fig. 3D). Further analysis by chromatin immunoprecipitation (ChIP) revealed that NURR1 is associated with the *ATG7* and 12 promoters (Fig. 3E). Nurr1 was also associated with the Sp1 but not the ATG13 (negative control) promoter in a ChIP assay (Fig. 3E). We also subcloned the *ATG7* and *ATG12* promoter sequences into a luciferase reporter plasmid and after transfection into MiaPaCa2 cells the effects of NURR1 knockdown on luciferase activity was determined. Knockdown of *NURR1* expression significantly decreased luciferase activity in both *AGT7 and AGT12* promoter-luciferase constructs (Fig. 3F) confirming that *ATG7* and *ATG12* are key autophagic genes regulated by NURR1. Therefore, the role of *NURR1* and *ATG7*/*ATG12* in gemcitabine resistance was further investigated.

### NURR1 Induces Autophagy, ATG7, and ATG12 in Pancreatic Cancer Cells

**FIGURE 4:**
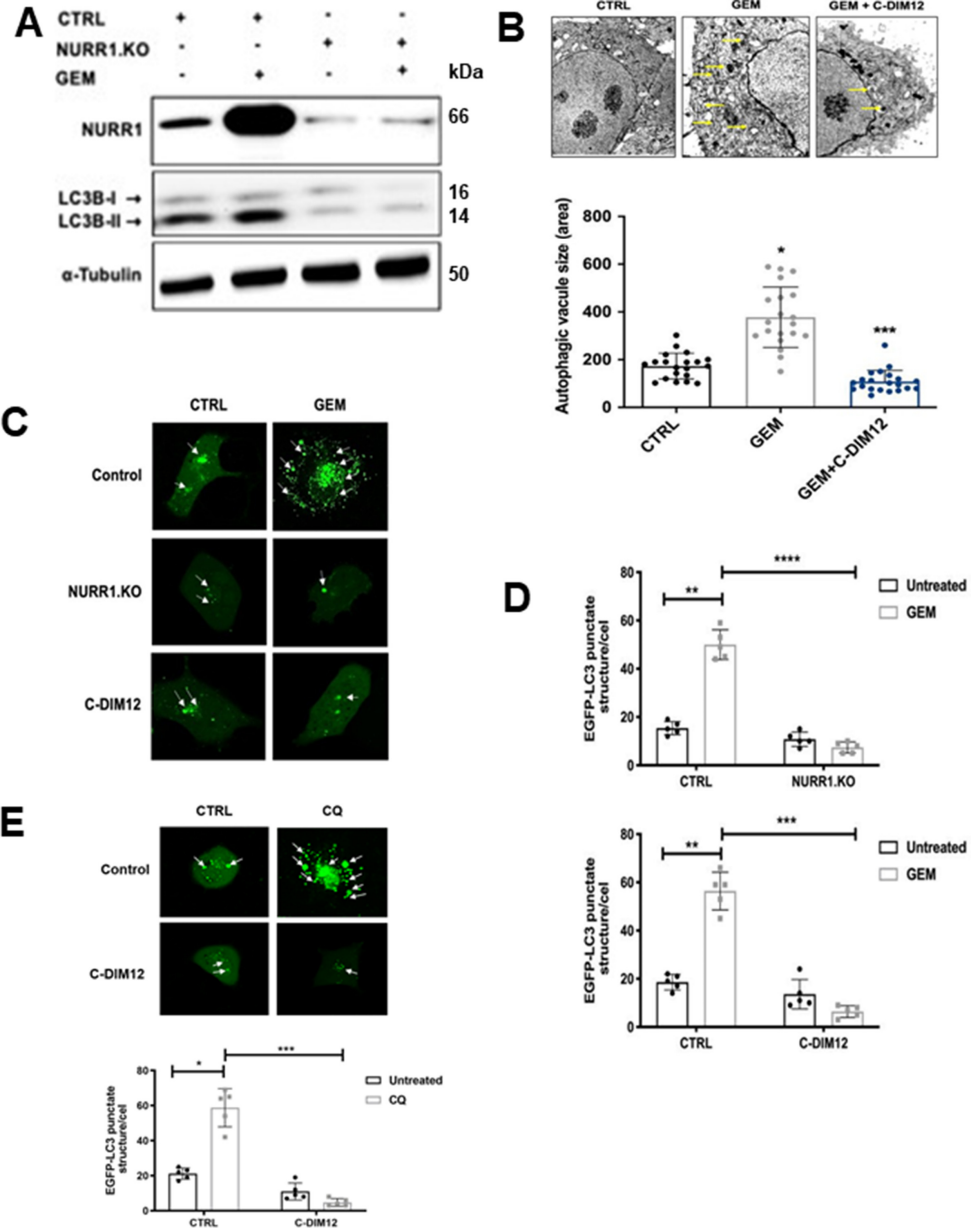
*NURR1* induces autophagy in pancreatic cancer. **A,** Immunoblot analysis of *NURR1* and LC3-I and LC3-II in *NURR1.KO* cells compared with control (empty vector; EV) MiaPaCa2 cells with α-tubulin as loading control. **B,** MiaPaCa2 cells were treated with gemcitabine (GEM) or C-DIM12 and autophagosomes were observed using transmission electron microscopy. The yellow arrow head indicates the autophagosomes and autolysosomes. The size of autophagosome was analyzed by measuring the area of autophagic vacuoles. **C,** Confocal microscopy images of MiaPaCa2 cells for EGFP-LC3 punctate staining in control (EV) and *NURR1.KO* cells treated with gemcitabine and C-DIM12 for 24 hours; white arrows indicate punctate EGFP-LC3 structures. **D,** As a control experiment, we show that chloroquine induces punctate screening. **E,** Bar graphs representation of the average EGFP-LC3 punctate in the cells (*, *P* < 0.05; **, *P* < 0.01; ****, *P* < 0.0001; three independent experiments performed, and at least 10 images per slide were analyzed for each condition). Scale bar, 10 μm. Magnification, 40×.

**FIGURE 5:**
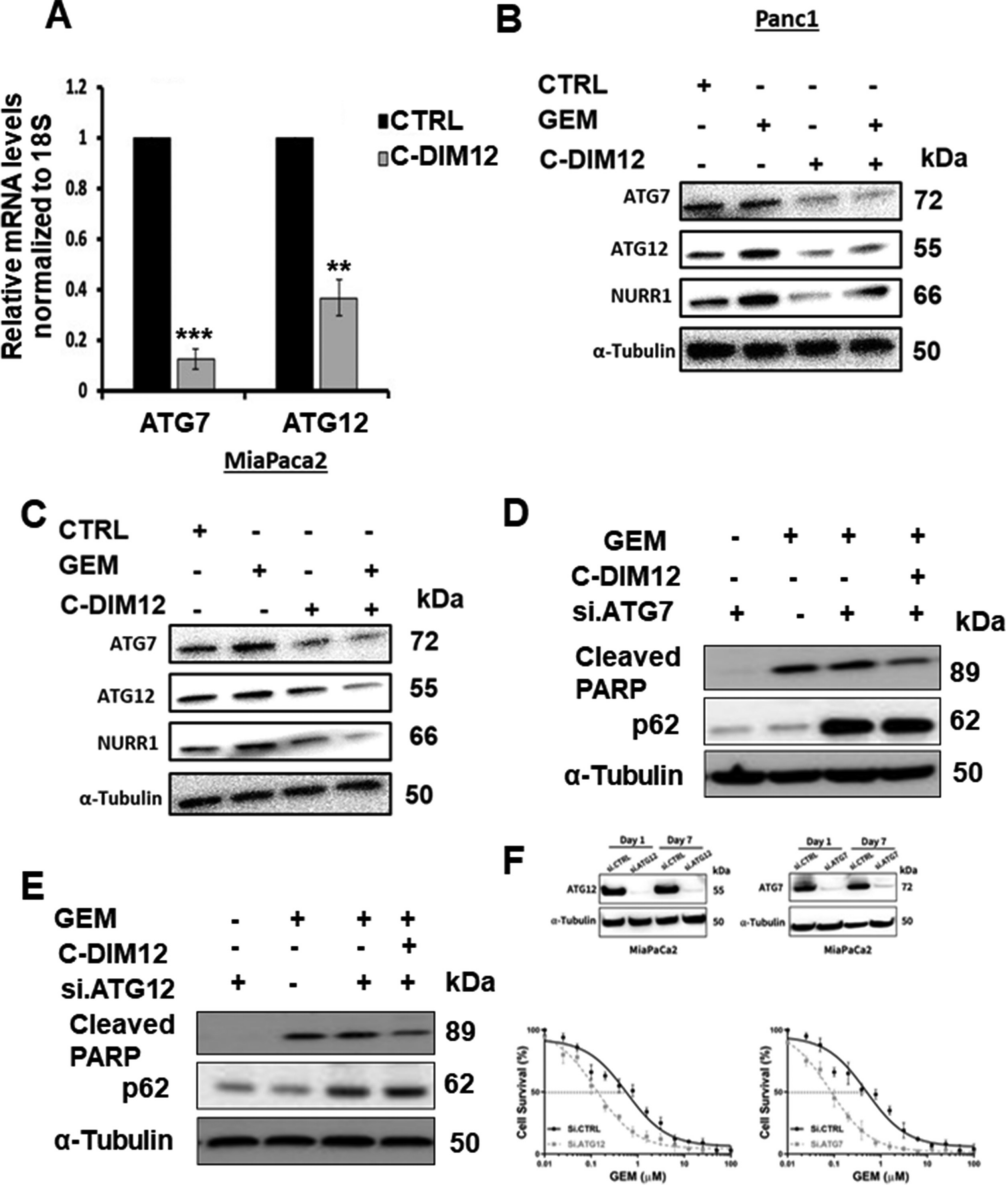
*NURR1* regulates the expression of *ATG7* and *ATG12.***A,** qPCR analysis of *ATG7* and *ATG12* expression after treatment with C-DIM12 (**, *P* < 0.01; ***, *P* < 0.001). **B,** Immunoblot analysis of *ATG7* and *ATG12* and cleaved PARP in gemcitabine (GEM) and C-DIM12–treated MiaPaCa2 cells. **C,** Immunoblot analysis of *ATG7* and *ATG12* in gemcitabine and C-DIM12–treated Panc1 cells. **D** and **E,** Immunoblot analysis of cleaved PARP and p62 in the presence of si.*ATG7* or si.*ATG12* MiapaCa2 cells in the presence or absence of treatment with gemcitabine, C-DIM12, and combination of both. α-Tubulin serves as a loading control. **F,** Effects of ATG7 and ATG12 knockdown on gemcitabine-induced survival of MiaPaca2 cells was determined as outlined in Fig. 2F. Each data point represents the mean of four independent measurements.

Autophagy is a cellular self-degradation process that reduces cellular damage in response to stressful conditions and the link between *NURR1* expression and autophagy was further investigated in MiaPaCa2 cells treated with gemcitabine. Figure 1D and E show that gemcitabine induced *NURR1* expression in Panc1 and MiaPaCa2 cells and this is also observed in Fig. 4A where gemcitabine-induced NURR1 expression is accompanied by an increase in the autophagic marker *LC3B-II*, whereas in NURR-KO cells ± gemcitabine, low levels of LC3B-II were expressed (Fig. 4A). Next, electron microscopy supports the immunoblot data showing that gemcitabine induced autophagy, which is characterized by autophagosomes and autophagic compartments; however, treatment with gemcitabine in combination with the NURR1 antagonist C-DIM12 reduced evidence for autophagy (Fig. 4B). Confocal microscopy also demonstrated that gemcitabine induced autophagy and that knockdown of *NURR1* or treating the cells with C-DIM12 significantly reduced autophagy marked by decreased punctate staining of LC3-II (Fig. 4C). The bar graph representation revealed that LC3-II punctate staining was decreased 2–3-fold when expression of *NURR1* was inhibited (Fig. 4D) and as a control we show that chloroquine increased LC3-II punctate staining (Fig. 4E). These data suggest that *NURR1* is essential for induction of autophagy in pancreatic cancer cells. We next investigated the effects of the *NURR1* antagonist C-DIM12 on expression of *ATG7* and *ATG12* in MiaPaCa2 cells and showed that C-DIM12 decreased expression of *ATG7* and *ATG12* mRNA levels (Fig. 5A). Treatment with C-DIM12 in combination with gemcitabine significantly downregulated expression of *ATG7* and *ATG12* in immunoblot analysis in MiaPaCa2 and Panc1 cells (Fig. 5B and C) and in the latter cell line, C-DIM12 decreased Nurr1 expression. Gemcitabine alone induced PARP cleavage in MiaPac2 cells (Fig. 5D and E), whereas knockdown of ATG7 or ATG12 did not induce this response. In contrast, gemcitabine alone and gemcitabine plus knockdown of ATG7 (Fig. 5D) or ATG12 (Fig. 5E) induced PARP cleavage and p62, and these responses were not enhanced by C-DIM12. The unexpected synergistic interactions of gemcitabine plus knockdown of ATG7 and ATG12 on induction of p62 are being further investigated. We also observed that silencing of ATG7 or ATG12 enhanced the cytotoxicity of gemcitabine (Fig. 5F) and these results are consistent with enhanced gemcitabine cytotoxicity after treatment with C-DIM12 or knockdown of Nurr1 (Fig. 2F).

### *In Vivo* Confirmation of NURR1/ATG7/ATG12 Axis

**FIGURE 6:**
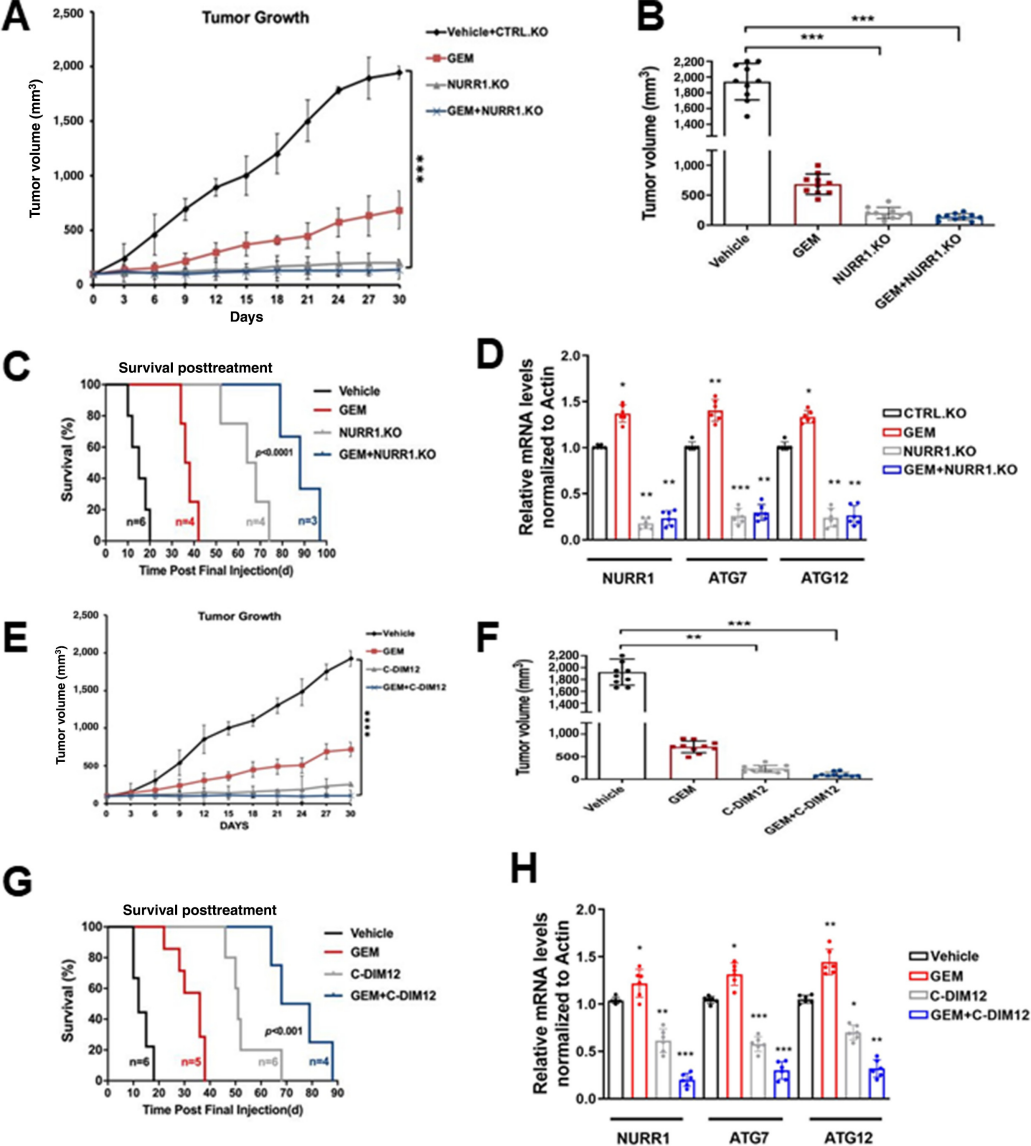
Gemcitabine (GEM) in combination with C-DIM12 inhibits tumor growth *in vivo*. **A,** Tumor growth curves of MiaPca2 CTRL and *NURR1*.KO xenografts in nude mice treated with vehicle, gemcitabine, or combined *NURR1*.KO and gemcitabine for 30 days, when control tumors reached 100 mm^3^ in size. Tumor size was assessed every 3 days using digital calipers. (*n* = 5 per group, two tumors per mouse; ***, *P* < 0.001). Mean and SD are shown. **B,** Average tumor volume of MiaPaca2 (vehicle, gemcitabine, *NURR1.KO*, and combination of both) xenografts at the end of the experiment (day 30; *n* = 10 tumors per group; ***, *P* < 0.001). **C,** Survival was assessed after gemcitabine cessation. Mice were removed from the group when tumors achieved a volume of 1,500 mm^3^. Statistical significance was determined by log-rank test. **D,** Tumor RNA was extracted from vehicle or combination of gemcitabine and *NURR1.KO* xenografts and analyzed for mRNA expression of *NURR1*, *ATG7*, or *ATG12*. Normalized values are shown as mean ± SEM (*n* = 6 tumors per group; **, *P* < 0.01). **E,** Tumor growth curves of MiaPca2 xenografts in nude mice treated with vehicle, gemcitabine, C-DIM, or combined for 30 days (*n* = 5 per group, two tumors per mouse; ****, *P* < 0.0001). **F,** Average tumor volume of MiaPacA2 (vehicle, gemcitabine, C-DIM, and combination of both) xenografts at the end of the experiment (day 30; *n* = 10 tumors per group; **, *P* < 0.01; ***, *P* < 0.001). **G,** Survival was assessed after treatment cessation. Mice were removed from the group when tumors achieved a volume of 1,500 mm^3^. Statistical significance was determined by log-rank test. **H,** Tumor RNA was extracted from vehicle or combination of gemcitabine and C-DIM xenografts and analyzed for mRNA expression of *NURR1*, *ATG7*, or *ATG12*. β-Actin was used as normalization control. Normalized values are shown as mean ± SEM (*n* = 6 tumors per group; *, *P* < 0.05; **, *P* < 0.01).

In athymic nude mice bearing MiaPaCa2 cells (Ctrl and *NURR1*-KO), gemcitabine alone decreased tumor volume (Fig. 6A and B), and both tumor volumes and weights were decreased in mice bearing NURR1-KO cells alone and after treatment with gemcitabine. Compared with the vehicle-treated group, the combination of gemcitabine, and *NURR1* knockdown, also extended the median survival of the mice from 20 to 97 days (Fig. 6C). Both *ATG7* and *ATG12* mRNA levels were lower in tumors derived from mice bearing *NURR1*-KO cells and treated with gemcitabine compared with control cells (Fig. 6D). A comparable set of data were obtained in tumors from mice bearing wild-type MiaPaCa2 cells and treated with vehicle, gemcitabine, C-DIM12 and their combination. Both gemcitabine and C-DIM12 and their combination inhibited tumor growth (Fig. 6E and F) to a similar extent; however, posttreatment survival (Fig. 6G) shows that gemcitabine plus C-DIM12 was more effective than either compound alone. Gemcitabine plus C-DIM12 also inhibited *ATG7* and *ATG12* mRNA levels (Fig. 6H) and this corresponds to results observed in the *in vitro* studies (Fig. 5).

### Prognostic Significance of ATG7 and ATG12 and Association of NURR1 and ATG7 and ATG12 Expressions in Clinical Specimens

**FIGURE 7:**
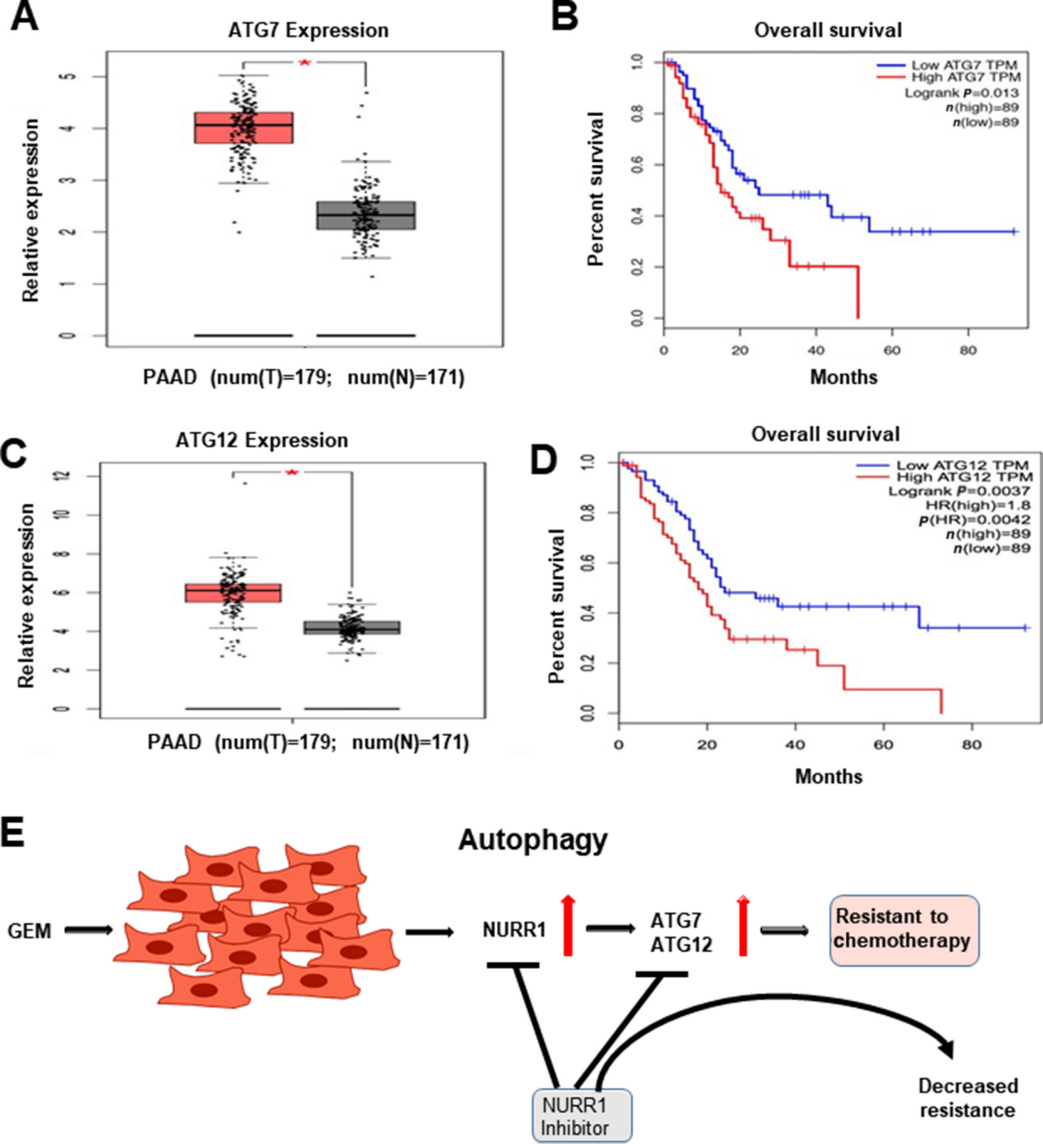
Prognostic significance of *ATG7* and *ATG12* in PDA. **A,***ATG7* expression in PDA patient tumor samples as compared with normal pancreas tissue using TCGA database (*, *P* <0.05). **B,** Kaplan–Meier analysis to demonstrate the prognostic significance of *ATG7* in PDA patient (*P* = 0.013). **C,***ATG12* expression in PDA patient tumor samples as compared with normal pancreas tissue using TCGA database (*, *P* <0.05). **D,** Kaplan–Meier analysis to demonstrate the prognostic significance of *ATG12* in PDA patient (*P* = 0.0042). **E,** Model for the role of NURR1 in gemcitabine (GEM) drug resistance in PDAC and effects of NURR1 antagonists to decrease drug resistance.

To study the prognostic significance of *ATG7* and *ATG12* in PDA, we examined TCGA, which showed a significant increase in expression of *ATG7* in pancreatic tumors (T = 179) compared with normal tissue samples (*n* = 171; Fig. 7A). Kaplan–Meier analysis survival curve showed that high expression of *ATG7* correlated with poor survival rate of patients with PDA (Fig. 7B). This analyses also demonstrated a significant increase in the expression level of *ATG12* in pancreatic tumors, (T = 179) compared with normal tissue samples (N = 171; Fig. 7C) and Kaplan–Meier analysis shows that high expression of *ATG12* corresponds to poor patient survival rate (Fig. 7D). We also investigated the correlation between the expression of *NURR1* and *ATG*s (*ATG7* and *ATG12*) in pancreatic tumor samples by performing correlation analysis. *NURR1* expression was strongly correlated with *ATG7* expression (*R* = 0.2, *P* = 6.2e-05; Supplementary Fig. S2C) and *ATG12* expression (R = 0.31, *P* = 2e-05; Supplementary Fig. S2D) in PDA patient samples. Together, these data indicate prognostic significance of *ATG7* and *ATG12* in PDA and their correlation with *NURR1* expression. These data coupled with mechanistic studies suggest a role for *NURR1* in gemcitabine-induced resistance, which is linked to enhanced autophagy and the potential clinical applications of combination therapies using gemcitabine and NURR1 antagonists (Fig. 7E). The linkage between NURR1 and ATG7/ATG12 has been clearly demonstrated; however, the possible interactions with other important autophagic factors (e.g., TFEB) has not been determined and is currently been investigated.

## Discussion

PDA is usually detected in later stages and patients with PDA have low survival rates and current treatment options are limited in their effectiveness. Improvements in PDA patient survival will require development of validated biomarkers that appear early in the formation of these tumors and also new mechanism-based drugs and drug combinations that increase efficacy and decrease drug resistance. We initially examined the TCGA database and observed that expression of the orphan nuclear receptor *NURR1* was more highly expressed in pancreatic tumors compared with the normal pancreas and patients with pancreatic cancer expressing high levels of *NURR1* exhibited decreased survival (Fig. 1) and this was similar to our recent studies on the expression and prognostic value of NURR1 in glioblastoma (15). The functions of *NURR1* have been investigated in multiple tumors (9, 33–38) and results of knockdown or overexpression studies have characterized this receptor as a prooncogenic factor that regulates cancer cell proliferation, survival, migration, and invasion. Although an endogenous ligand for *NURR1* has not been identified, a recent study shows that the bis-indole–derived C-DIM12, a known *NURR1* ligand, acted as a receptor antagonist in glioblastoma cells and inhibited cell growth and invasion and increased apoptosis (15). Another report showed that overexpression of *NURR1* in squamous cell carcinoma cells increased resistance to 5-FU treatment (17) suggesting a possible role for *NURR1* in drug resistance. Gemcitabine has replaced 5-FU for treatment of pancreatic cancer and based on the prognostic and functional characteristics of *NURR1* in cancer cells this study focused on determining the functions of *NURR1* in pancreatic cancer and its role in gemcitabine resistance.

Knockout of *NURR1* in pancreatic cancer cells or treatment with C-DIM12–induced apoptosis and inhibited proliferation (Fig. 2). Moreover, in athymic nude mice bearing MiaPaCa2 (*NURR1*^+/+^) or *NURR1*-KO cells, it was clear that loss of *NURR1* or treatment with C-DIM12 inhibited tumor growth and enhanced survival (Fig. 6). These results confirmed the prooncogenic activity of *NURR1* in pancreatic cancer cells as previously observed in other cancer cell lines (15) and demonstrated that the *NURR1* antagonist C-DIM12 was an effective anticancer agent that blocked *NURR1*-mediated responses.

We also investigated the role of *NURR1* in drug resistance and observed that treatment of pancreatic cancer cells with gemcitabine resulted in induction of *NURR1* (Fig. 1). Thus, although gemcitabine alone induced apoptosis and inhibited growth of pancreatic cancer cells and tumors (Figs. 1 and 6) this was accompanied by induction of *NURR1*, which exhibits tumor promoter–like activity. This counterintuitive effect of gemcitabine could be a component of drug resistance and this was confirmed in combination treatments of cells and mice with gemcitabine alone and in combination with NURR1 knockdown or receptor antagonist (C-DIM12; Figs. 1 and 6). Results of the *in vitro* and *in vivo* effects of these combination therapies are complementary and demonstrate the *NURR1* inactivation or treatment with C-DIM12 enhanced the effectiveness of gemcitabine thus demonstrating that induction of *NURR1* by gemcitabine is associated with drug resistance.

The mechanism of *NURR1* as a drug-resistant factor was further investigated by RNA-seq in wild-type and *NURR1*-KO cells and this resulted in identification of several genes/pathways regulated by *NURR1*; however, the major pathway was associated with changes in expression of genes involved in autophagy (Fig. 3; refs. 39–43). Because autophagy has previously been linked to cytoprotection in colon cancer cells (21), we examined this gene set and identified two genes, namely *ATG7* and *ATG12*, that were not only regulated by *NURR1* (Fig. 5A and B) but also exhibit clinical characteristics similar to that observed for *NURR1* in patients with pancreatic cancer (Figs. 1 and 7). These results confirm that *NURR1* exhibits prooncogenic-like activity in pancreatic cancer cells and this receptor also plays a role in gemcitabine-induced drug resistance (Fig. 7E). These observations coupled with the anticarcinogenic effects of C-DIM12 suggest that the effectiveness of therapeutic regimens including gemcitabine for treatment of PDAC can be significantly enhanced using combination therapies that include a *NURR1* antagonist such as C-DIM12.

## Supplementary Material

Supplementary Data Supplementary text Figs. 1 and 2 Supplementary text Table S1 and S2.
