# Supplementary material for: Nuclear Receptor 4A2 (NR4A2/NURR1) Regulates Autophagy and Chemoresistance in Pancreatic Ductal Adenocarcinoma
Source: Cancer Res Commun. 2021 Nov 3;1(2):65–78. doi: 10.1158/2767-9764.CRC-21-0073 (PMC9109828; doi:10.1158/2767-9764.CRC-21-0073)
Supplement: Supplementary Data — Supplementary text Figs. 1 and 2 Supplementary text Table S1 and S2. [file crc-21-0073-s01.docx]

**Supplementary Data for:**

**Nuclear Receptor 4A2 (NR4A2/NURR1) Regulates Autophagy and Chemoresistance in Pancreatic Ductal Adenocarcinoma**

Mehrdad Zarei^1^*, Rupesh Shrestha^2^*, Sneha Johnson #^2^, Zuhua Yu^2,3^#, Keshav Karki^2^, Ali Vaziri-Gohar^1^, Jessica Epps^4^, Heng Du^5^, Larry Suva^2^, Mahsa Zarei^2,5^ and Stephen Safe^2^

^1^Department of Surgery, University Hospitals; Case Western University, School of Medicine, Cleveland, OH

^2^Department of Veterinary Physiology and Pharmacology, Texas A&M University, College Station, TX

^3^Henan University of Science and Technology, Luoyang, Henan Province, China, P.R.

^4^Department of Integrative Biosciences, College of Veterinary Medicine, Texas A&M University, College Station, TX

^5^Department of Medicine, Brigham and Women's Hospital and Harvard Medical School, Boston, MA

 * Contributed equally-First authors

# Contributed equally-Second authors

**Running Title:** *NURR1* regulates autophagy via regulation of *ATG7* and *12*

**Corresponding Author:**

Mahsa Zarei, PhD, Department of Veterinary Physiology and Pharmacology, College of Veterinary Medicine and biomedical Sciences, Texas A&M University, 4466 TAMU, College Station, TX 77843-4466 USA, Telephone: 979-458-8680, Email: mzarei@bwh.harvard.edu

Stephen H. Safe, Ph. D. Distinguished Professor, College of Veterinary Medicine & Biomedical Sciences, Department of Veterinary Physiology & Pharmacology, Texas A&M University, 4466 TAMU, College Station, TX 77843-4466 USA, Tel: 979-845-5988 / Fax: 979-862-4929, Email: [ssafe@cvm.tamu.edu](mailto:ssafe@cvm.tamu.edu)

**Disclosure of Conflicts of Interest:** There are no other conflicts of interests to declare.

**This PDF file includes:**

**Supplementary text Figs. 1 and 2**

**Supplementary text Table S1 and S2.**

**
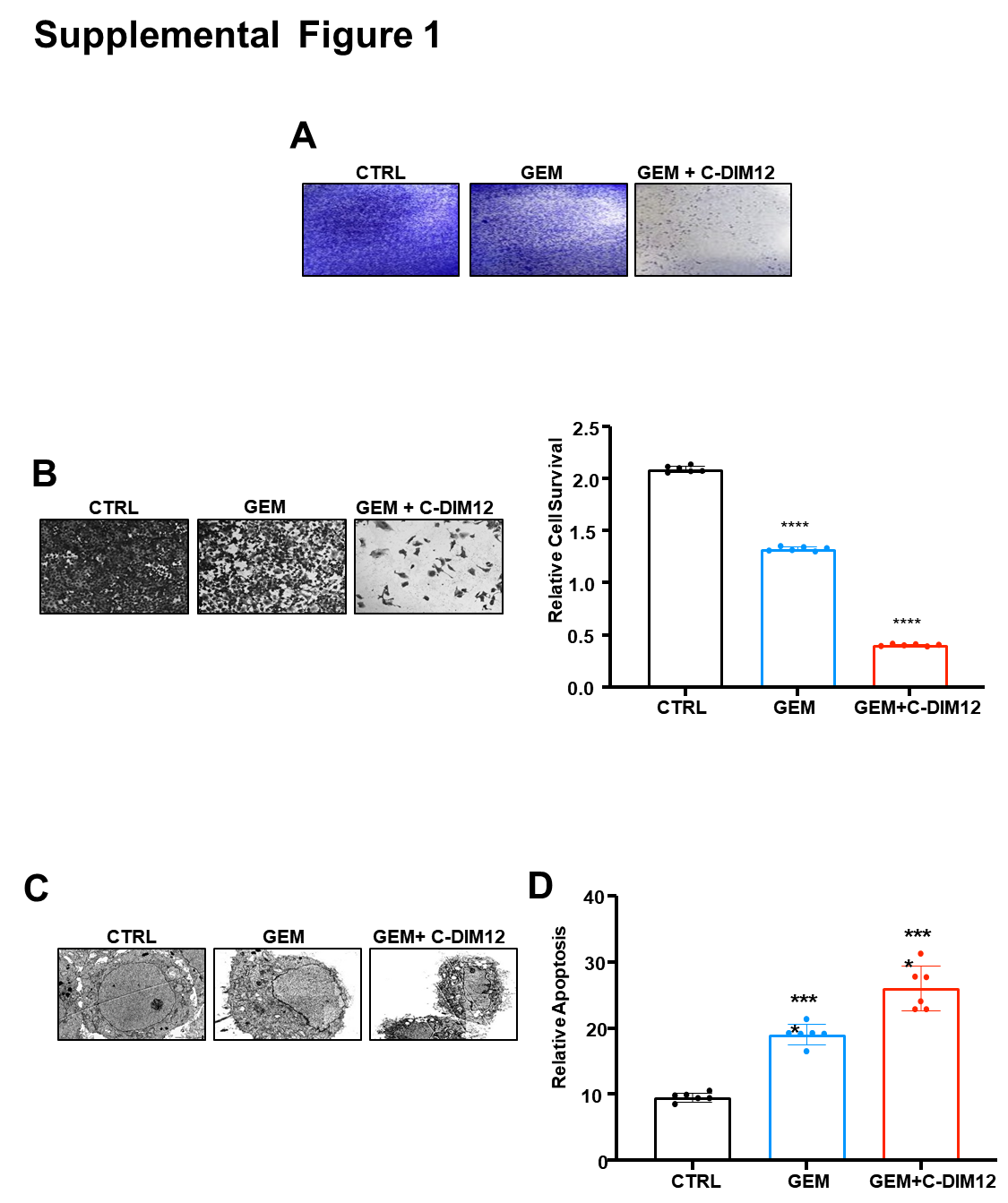
Supplemental Figure 1.** A) Images of crystal violet stained dishes Panc1 cells that were treated with vehicle control, gemcitabine (3 independent experiments performed). B) Phase contrast images and bar graph for percentage of cell survival in Panc1 cells after treatment with vehicle control, gemcitabine and C-DIM12, **** *p* < 0.0001, C) Panc1 cells were treated with gemcitabine or C-DIM12 and autophagosomes were observed using transmission electron microscopy, D) Apoptotic cell fraction was determined after treatment control, gemcitabine, or combination of GEM and C-DIM12 for 72hr. Each data point represents the mean ± SEM of three independent experiments, **** *p* < 0.0001).

**
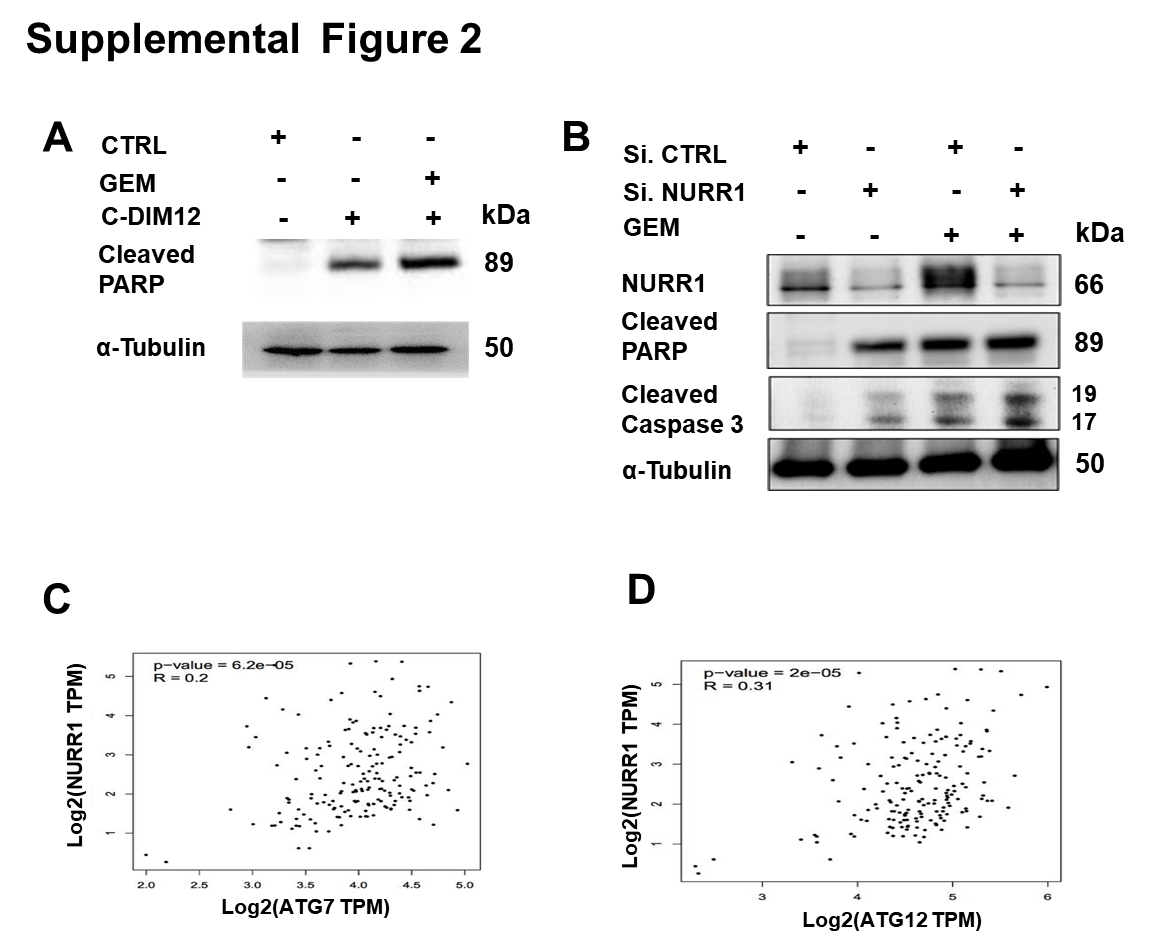
**

**Supplemental Figure 2.** A. Immunoblot analysis of Cleaved PARP in control, gemcitabine, C-DIM12 treated cells with α-Tubulin as loading control in Panc1, B) Immunoblot analysis of *NURR1*, Cleaved PARP, Cleaved Caspase 3 in siCTRL and si*NURR1* MiaPaCa2 cells, treated with gemcitabine. C. Correlation analysis of *NURR1* and *ATG7* expression in pancreatic tumor samples, D) Correlation analysis of the expression of *NURR1* and *ATG12* in pancreatic tumor samples. Pearson correlation coefficient (R^2^) and p-values are shown (**** p < 0.0001).

**Supplemental Table S1.** Summary of reagents, antibodies, immune staining sources, oligonucleotides and primers

| Reagents | Source | Identifier |
| --- | --- | --- |
| DMEM | Invitrogen | 11995-065 |
| Antibiotic | Invitrogen | 12450-062 |
| Fetal Bovine Serum (FBS) | Invitrogen | 26140-079 |
| Lipofectamine 2000 | Invitrogen | 11668-019 |
| Trypsin | Invitrogen | 25200-056 |
| Mithramycin | Cayman Chemical | 11434 |
| Chemiluminescence reagents | EMD Millipore | WBKLS0500 |
| PVDF membrane | Biorad | 1620184 |
| SDS Solution | National Diagnostic | EC-874 |
| Protogel (30%) | National Diagnostic | EC-890 |
| Protein Ladder | Biorad | 1610375 |
| Collagenase IV | Worthington Biochemical Corporation | LS004188 |
| DNase I | Zymo Research | E1010 |
| BD Perm/wash | BD Biosciences | 51-2091KZ |
| RBC Lysis buffer | eBioscience | 00-4333-57 |

| Antibodies | Source | Identifier |
| --- | --- | --- |
| PD-L1 (Human Specific) | Cell Signaling Technology | 13684s |
| PD-L1 (Mouse Specific) | R&D System | Mab90781 |
| Secondary anti-Rabbit-HRP | Cell Signaling Technology | 7074s |
| Secondary anti-Mouse-HRP | Cell Signaling Technology | 7076s |
| NR4A1 | Abcam | ab109180 |
| Sp1 | Abcam | ab13370 |
| Sp1 | Santacruz Biotechnology | sc-17824X |
| Β-actin | Sigma Aldrich | A5316 |
| RNA Pol II | Active motif | 102660 |
| Anti-mouse CD45 | BD Biosciences | 557659 |
| Anti-mouse CD3 | BD Biosciences | 560591 |
| Anti-mouse CD4 | BioLegend | 100541 |
| Anti-mouse CD8 | BD Biosciences | 557688 |
| Anti-mouse CD244.1 (2B4) | BD Biosciences | 745953 |
| Anti-mouse TIGIT | BD Biosciences | 565168 |
| Anti-mouse CD366 (TIM3) | BD Biosciences | 747622 |
| Anti-mouse CD279 (PD-1) | Invitrogen | 61-9985-82 |
| Anti-mouse T-bet | BioLegend | 644817 |
| Anti-mouse CD16/32 (Fc blocker) | Invitrogen | 14-0161-82 |
| Anti-mouse Foxp3 | BD Biosciences | 562996 |
| Anti-mouse CD25 | BioLegend | 102049 |

| Resources (Assay kit) | Source | Identifier |
| --- | --- | --- |
| Live/dead cell stain | Invitrogen | L10119 |
| Foxp3/ Transcription Factor Staining Buffer Set | Invitrogen | 00-5523-00 |
| Compensation beads | Invitrogen | 01-2222-42 |
| DNA/RNA extraction kit | Zymo Research | 11309, R1055 |
| MojoSort Mouse CD8 T Cell Isolation Kit | BioLegend | 480007 |

| Oligonucleotides | Source | Identifier |
| --- | --- | --- |
| PD-L1 ChIP Primer (Human)  Forward GAAGGTCAGGAAAGTCCAAC  Reverse TCGGGAAGCTGCGCAGAACT | Reference #25 | N/A |
| PD-L1 ChIP Primer (Mouse)  Forward CGAGCTTCAACCAATCAGCG Reverse GAATTTGCGGTTCTGTCCCG | Reference #25 | N/A |
| GAPDH (Mouse)  Forward AGGTCGGTGTGAACGGATTTG  Reverse GGGGTCGTTGATGGCAACA | IDT | 1 |
| NR4A1 (Mouse)  Forward ATGCCTCCCCTACCAATCTTC  Reverse CACCAGTTCCTGGAACTTGGA | IDT | 1 |
| TOX (Mouse)  Forward TGCCTGGACCCCTACTATTG  Reverse CTGGCTGGCACATAGTCCTG | IDT | 2 |
| TOX2 (Mouse)  Forward AGCAGAAACAGGCGTATAAGAGG  Reverse GTACATGGGCTGCTTGGGTG | IDT | 2 |
| TBx21 (T-bet) (Mouse)  Forward CAACAACCCCTTTGCCAAAG  Reverse TCCCCCAAGCATTGACAGT | IDT | 3 |
| NFAT1 (Mouse)  Forward GTGCAGCTCCACGGCTACAT  Reverse GCGGCTTAAGGATCCTCTCA | IDT | 4 |
| IFN-γ (Mouse)  Forward GGATGCATTCATGAGTATTGC  Reverse GTGGACCACTCGGATGAG | IDT | 5 |
| Granzyme-B (Mouse)  Forward CCACTCTCGACCCTACATGG  Reverse GGCCCCCAAAGTGACATTTATT | IDT | 5 |
| Perforin (Mouse)  Forward GAGAAGACCTATCAGGACCA  Reverse AGCCTGTGGTAAGCATG | IDT | 6 |
| siSP1 (Human) | Sigma Aldrich | SASI_Hs02_00333289 (1)  SASI_Hs02_00070994 (2) |
| siSP1 (Mouse) | Sigma Aldrich | SASI_Mm01_00145222 (1)  SASI_Mm01_00145223 (2) |
| siNR4A1 (Human) | Sigma Aldrich | CAGUGGCUCUGACUACUAU (1)  GAGAGCUAUUCCAUGCCUA (2) |
| siNR4A1 (Mouse) | Sigma Aldrich | SASI_Mm01_00077215 (1)  SASI_Mm01_00077216 (2) |
| PD-L1 (Human) | Sigma Aldrich | GGUCAACGCCACAGCGAAUUU (1)  CCUACUGGCAUUUGCUGAACGCAUU (2) |
| PD-L1 (Mouse) | Sigma Aldrich | SASI_Mm01_00062675 (1)  CCCACAUAAAAAACAGUUGTT (2) |
| Scrambled siRNA | Sigma Aldrich | CGU ACG CGG AAU ACU UCG A |


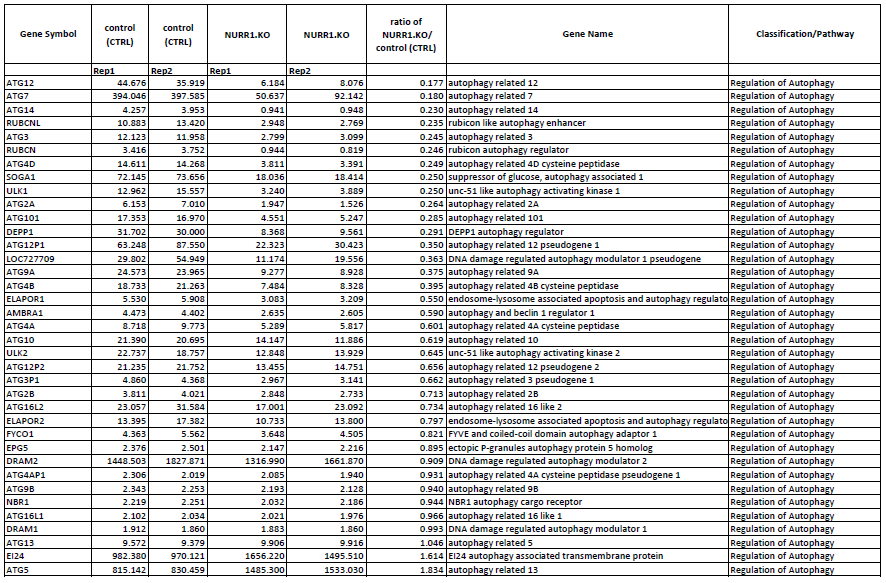
**Supplemental Table S2: Summary of NR4A2 regulated genes that play a role in autophagy**
